# From Binary to Multi-Class Classification: A Two-Step Hybrid CNN-ViT Model for Chest Disease Classification Based on X-Ray Images

**DOI:** 10.3390/diagnostics14232754

**Published:** 2024-12-06

**Authors:** Yousra Hadhoud, Tahar Mekhaznia, Akram Bennour, Mohamed Amroune, Neesrin Ali Kurdi, Abdulaziz Hadi Aborujilah, Mohammed Al-Sarem

**Affiliations:** 1 LAMIS Laboratory, Larbi Tebessi University, Tebessa 12002, Algeria; yousra.hadhoud@univ-tebessa.dz (Y.H.); tahar.mekhaznia@univ-tebessa.dz (T.M.); mohamed.amroune@univ-tebessa.dz (M.A.); 2 College of Computer Science and Engineering, Taibah University, Medina 41477, Saudi Arabia; nkordi@taibahu.edu.sa; 3 Department of Management Information Systems, College of Commerce & Business Administration, Dhofar University, Salalaha 211, Oman; aborujilah@du.edu.om; 4 Department of Information Technology, Aylol University College, Yarim 547, Yemen; mohsarem@gmail.com

**Keywords:** chest diseases, X-rays, convolutional neural networks, vision transformers, classification

## Abstract

Background/Objectives: Chest disease identification for Tuberculosis and Pneumonia diseases presents diagnostic challenges due to overlapping radiographic features and the limited availability of expert radiologists, especially in developing countries. The present study aims to address these challenges by developing a Computer-Aided Diagnosis (CAD) system to provide consistent and objective analyses of chest X-ray images, thereby reducing potential human error. By leveraging the complementary strengths of convolutional neural networks (CNNs) and vision transformers (ViTs), we propose a hybrid model for the accurate detection of Tuberculosis and for distinguishing between Tuberculosis and Pneumonia. Methods: We designed a two-step hybrid model that integrates the ResNet-50 CNN with the ViT-b16 architecture. It uses the transfer learning on datasets from *Guangzhou Women’s and Children’s Medical Center* for Pneumonia cases and datasets from *Qatar and Dhaka (Bangladesh) universities* for Tuberculosis cases. CNNs capture hierarchical structures in images, while ViTs, with their self-attention mechanisms, excel at identifying relationships between features. Combining these approaches enhances the model’s performance on binary and multi-class classification tasks. Results: Our hybrid CNN-ViT model achieved a binary classification accuracy of 98.97% for Tuberculosis detection. For multi-class classification, distinguishing between Tuberculosis, viral Pneumonia, and bacterial Pneumonia, the model achieved an accuracy of 96.18%. These results underscore the model’s potential in improving diagnostic accuracy and reliability for chest disease classification based on X-ray images. Conclusions: The proposed hybrid CNN-ViT model demonstrates substantial potential in advancing the accuracy and robustness of CAD systems for chest disease diagnosis. By integrating CNN and ViT architectures, our approach enhances the diagnostic precision, which may help to alleviate the burden on healthcare systems in resource-limited settings and improve patient outcomes in chest disease diagnosis.

## 1. Introduction

*Chest diseases*, also known as thoracic diseases, encompass a wide range of medical illnesses that affect organs and structures inside the chest cavity. Millions of people around the world suffer from chest disorders which cause discomfort and hinder daily activities [1]. There are more than 200 types of chest diseases, including Pneumonia, Tuberculosis, pleural effusion, cardiomegaly, lung cancer, etc. According to the *World Health Organization* (WHO), the *Chronic Obstructive Pulmonary Disease* (COPD) remains globally the third leading cause of death [2]. It resulted in 3.5 million deaths in 2019 (“Chronic obstructive pulmonary disease (COPD)”, 2023). In 2023, 1.25 million people worldwide died from Tuberculosis (TB) [3]. In 2022, the United States reported 8300 TB cases and over 13 million people with latent TB infection [4]. Similarly, other countries faced significant TB burdens. India has approximately 2.95 million new TB cases, while Indonesia and China reported around 824,000 and 746,000 cases, respectively. South Africa and Nigeria had about 328,000 and 452,000 new cases, respectively [5]. Pneumonia claims a child every 43 s, making it the most significant infectious cause of death in this age group [6].

Diagnosing chest diseases typically entails physical examination combined with laboratory tests or biopsies; however, imaging studies, particularly chest X-rays (CXRs), are the most commonly used as diagnostic tools due to its fastness. Examining CXR images remains a challenge in regard to its subjective nature of interpretation, poor image quality, and low lighting conditions. Moreover, the similar patterns of many thoracic disorders lead to frequent misinterpretations. These challenges emphasize the need for developing *Computer-Aided Diagnosis* (CAD) systems for the early detection and accurate diagnosis of chest diseases.

CAD systems have proven to be highly effective in detecting and classifying chest diseases. With their capacity in extracting complex features, they excel at performing precise analyses of medical images, leading to new avenues for improving patient care and diagnostic accuracy. Consequently, CAD systems have become crucial tools in contemporary healthcare [7]. *Deep learning*, particularly (CNNs), is predominantly used for disease detection and classification. They achieve state-of-the-art results due to their robustness in handling variations in CXR images and their ability to capture hierarchical patterns and structures. However, CNNs primarily analyze the correlation between spatial adjacent pixels, making it difficult to determine relationships between distant pixels. Additionally, the convolution operation causes information loss and leads to overfitting, resulting in poor generalization. Accordingly, recent studies have focused on transformers [8], especially vision transformers (ViTs) [9]. Their architecture consists of a self-attention mechanism and a feed-forward neural network. The related key idea is to treat a given image as a series of patches, similar to how a sentence is treated in NLP. The self-attention mechanism used by ViTs allows for the comprehension of relationships between different regions of an image. *Transformers* are able to process sequences of data, which is advantageous in cases where the context of the whole image is important. In their first appearance, transformers were used in the field of *Natural Language Processing* (NLP), such as text generation, translation, etc. [10]. The groundbreaking results achieved in NLP prompted researchers to take advantage of this success in other fields, such as computer vision.

In this study, we focused on two critical chest diseases, namely Pneumonia and Tuberculosis. Both diseases appear in various forms, impacting different parts of lungs. They manifest with overlapping features in CXR images as pulmonary infiltrates, cavitation air bronchograms, and pleural effusion. This inhibits the distinction between them, hence leading to ambiguous diagnosis, especially in settings with a shortage of trained radiologists. On the other hand, CXR images are more cost-effective compared to the other modalities of early detection and treatment. Also, X-rays are non-invasive compared to laboratory tests such as *Polymerase Chain Reaction* (PCR) and biopsies, which seem painful. They have a risk of complication and call for specific knowledge and equipment. For this reason, we propose a hybrid model that benefits from the power of both CNNs and ViTs. It operates in a parallel manner, ensuring the early and accurate detection of diseases. CNNs are well known for their capacity to evaluate visual data by identifying hierarchical patterns and structures within images. ViTs, on the other hand, allow for capturing relationships between different regions of an image, leading the user to easily understand the global context of the image.

Hence, the main contribution of this study is illustrated by the following:

- The application of feature-level fusion to interpret complex features of X-ray images, especially overlapping characteristics.
- Supporting the parallelism through the application of a refined attention mechanism to both ResNet-50 and ViT-b16 streams after the feature extraction step. This attention mechanism enables the model to prioritize features consistently across diverse samples.
- Providing an effective solution to the significant challenge of data imbalance by combining oversampling and strategic augmentation techniques.

The rest of this paper provides a set of related works in Section 2, followed by a presentation of the proposed approach in Section 3. The experimental process is shown in Section 4, along with an analysis and discussion in Section 5 and Section 6, respectively. Finally, we conclude our work in Section 7, specifying future perspectives.

## 2. Literature Review

In this section, we explore various advancements and methodologies employed in recent studies focusing on CNNs, ViTs, and hybrid models that leverage the strength of both architectures.

### 2.1. Convolutional Neural Networks

CNNs have gained significant attention for medical image classification tasks. Several studies have since built upon this approach. The authors of [11] developed a lightweight CNN named ChestX-ray 6 for the detection of multiple thoracic diseases, including COVID-19, Pneumonia, pleural effusion, lung opacity, and cardiomegaly. It uses a combination of various datasets. The issue of imbalanced data was addressed with data augmentation. The model was first used in the classification of six kinds of diseases and then as a pretrained model for a binary classification of normal and Pneumonia CXR images. It achieved an accuracy of 80% for the first task and an accuracy of 97.94% with a recall of 98% for the binary classification. Similarly, Ref. [12] proved that their proposed lightweight CNN for epidemic lung disease prediction outperformed six pretrained CNNs, achieving an accuracy of 98.56%. In a previous study [13], we proposed a hybrid model based on a shallow CNN and XGboost model to detect and differentiate between viral and bacterial Pneumonia, achieving an accuracy of 96% for binary classification and 87% for multi-class classification.

A fine-tuned VGG-19 designed by [14] was used for multi-class chest disease classification, assessing lung cancer, lung opacity, Pneumonia, Tuberculosis, and COVID-19. It achieved an accuracy of 93.75%. The authors of [15] fine-tuned ResNet-50, VGG-16, and Inception-V3 to detect COVID-19 from CXR images using multiple combined datasets. The best performance was attained by VGG-16 with an accuracy of 98.30%. However, In Ref. [16], Authors classified COVID-19, non-COVID-19, viral Pneumonia, bacterial Pneumonia, and normal CXR images using a fine-tuned AlexNet model, which yield an accuracy of 93.42%. In [17], the authors trained a CNN model by applying transfer learning to differentiate between viral Pneumonia, bacterial Pneumonia and CXR images. This model achieved an accuracy of 92.8%. H. Malik et al. in [18] classified ten different chest diseases by proposing a novel fusion model with deep convolutional neural networks (DCNNs), following three essential steps: the segmentation of CXR images to isolate the lung region using the Info MGAN network [19], feature extraction using a hand-crafted technique, and the fusion of these feature, which were then fed to the DCNNs. Various machine learning models were used as the last layer of the DCNNs, achieving an accuracy of 98.20%.

In [20], authors identified chest abnormalities using the Vin Dr-CXR dataset [21] by proposing a model based on YOLO V5 for anomaly localization and ResNet50 for the classification, achieving a performance of 80% in terms of Mean Average Precision (MAP) and an F1-score of 76%. In [22], the authors proposed two models for COVID-19 detection using both CXR and Computer Tomography (CT) images. The first model stacked six pretrained models, and the second was a self-designed model based on ECA-Net and EfficientNet V2 using 10-fold cross validation. The results demonstrated that the difference between the performance of the two models was not significant, with an accuracy tending towards 99%.

### 2.2. Vision Transformers

Vision transformers are a type of neural networks, originally designed for NLP tasks, subsequently developed to benefit from its performance in computer-vision tasks such as classification [23], object localization [24] and segmentation [25]. ViTs leverage the self-attention mechanism, allowing the model to assess a relative performance of different parts within the input data and focus on critical features across the entire image. This task is achieved by splitting the image into smaller patches; each represents a small region of the original image. In so doing, the self-attention calculates the score for each pair of patches. The model, by focusing on informative patches, highlights the connection between a suspicious mass and surrounding healthy tissue in a medical X-ray.

In the field of medical images, ViTs have been used for many clinical purposes, including disease diagnosis and anomaly localization. The work in [26] used a new proposed pretrained Vision Outlooker model [27] (VOLO) for COVID-19 diagnosis, using a large dataset obtained by combining two available datasets [28,29]. The approach achieved an accuracy of 99.67%. For the same task, another fine-tuned ViT model was developed by M. Chetoui et al. [30]; they used the SIIM-FISABIO-RSNA COVID-19 dataset [31], achieving a performance of 96% in terms of sensitivity and specificity.

For multi-class classification, Krishan et al. [32] applied ViTs to classify COVID-19, Pneumonia, and normal CXR images, achieving an accuracy of 97.61%. The authors in [33] applied a ViT model for the classification of CXR images to normal lungs, diseased lungs, and COVID-19-affected lungs. To assess the impact of patches’ size on model performance, images were divided into various sizes, and a top accuracy of 95.36% was reached using a patch size of 32 × 32 pixels.

In [34], a deep model based on a pretrained swin-transformer was proposed for the multi-label classification of thoracic diseases using the chest X-ray-14 dataset [35]. It achieved a score of 81% in terms of Area Under the Curve (AUC). In [36], the authors applied a pretrained ViT model to the ChexPert dataset [37] and demonstrated performance gains above pretrained CNN-based VGG16 and ResNet architectures.

Moreover, Nasser, A.A. et al. [38] classified chest diseases using the merged datasets ChexPert [37] and VinDr-CXR [21] with a two-step approach; the first step involved classifying CXR images into three different classes, namely lung diseases, heart diseases, and normal. The second step involved classifying seven lung and heart pathologies as normal or abnormal by implementing two deep learning models (an ensemble deep CNN and a modified ViT). The modified ViT recorded a better performance with an AUC of 95.13% for the first step, and an average AUC of 99.26% and 99.57% for heart diseases and lung diseases, respectively, in the second step. In [39], the authors employed a ViT model to detect COVID-19 after segmenting the lung region with a U-Net model. The model achieved an AUC more than 95%. In [40], the authors adapted a ViT model to analyze CXR images by introducing an attention region selection module in the encoder layer of ViT. This technique enables the model to focus on most relevant regions. The model achieved an accuracy of 83.4% and a sensitivity of 86.3%. Another model named PneuNet was developed in [41] for Pneumonia and COVID-19 diagnosis. The model applies multi-head attention on channel patches rather than feature patches, achieving an accuracy of 94.96%.

### 2.3. Hybrid Models

Some works aim to benefit from the advantages of ViTs and CNNs by using a combination of them. The authors in [42] proposed an IEViT (Input-Enhanced Vision Transformer) model by introducing a CNN block in parallel with the ViT block to classify CXR images using four datasets containing different pathologies (COVID-19, Tuberculosis, Pneumonia). The CNN block embeds the input image and feeds it repeatedly to each transformer encoder layer by concatenating the image embedded to the input of each encoder layer. The experiments proved that the IEViT outperformed the original ViT and its variants in all the datasets, with a recall ranging between 93.5% and 100%.

Another hybrid model was proposed by [43] to identify Pneumonia based on CXR images. For the feature extraction step, an ensemble of pretrained CNNs was used, which was then fed to the transformer for the identification phase, achieving a best accuracy of 98.01%. In contrast, Ref. [27] suggested a hybrid model based on EfficientNet and ViT for the detection of Tuberculosis using a large dataset curated by combining several public datasets, recording an accuracy of 97.72%. To ensure the model’s performance, each of the EfficientNet and ViT models were tested separately, where the hybrid model achieved a better result.

In [44], the authors developed a model called VGG16-CoordAttention for automatic Tuberculosis detection, which entails adding a coordinated attention mechanism to the VGG16 architecture, achieving an accuracy of 92.73%. In a slightly different manner, Authors in [45] worked on TB detection using lateral CXR images obtained from [37,46] and thus, instead of frontal images. They built a model based on CNN and ViT which reached a performance of 91% in terms of the Matthews Correlation Coefficient (MCC). The authors in [47] developed a ResfEANet model for Tuberculosis detection based on a shallow ResNet. The model was trained from scratch without applying transfer learning. It achieved an accuracy of 97.59%. Additionally, a novel hybrid convolutional–transformer model named HydraViT was proposed by [48] for multi-classification. The model achieved an AUC of 83.8% across all the 14 pathologies.

The previous studies and numerous other existing works highlighted the impressive advancements in the use of CNNs and ViTs for thoracic disease prediction and classification. CNNs have been demonstrated to be effective in detecting abnormalities, achieving high accuracy rates. ViTs have provided a new dimension in medical imaging by capturing global context and long-range dependencies in images. Most of these studies focus on the use of CNNs, or ViTs separately, or using them in a sequential manner. The standalone CNNs [11,12] may have trouble in detecting long-range patterns and overlapped features. Pure ViTs [30] address this issue. However, the combination of both of them resulted in better performance, especially in the multi-class classification. The sequential hybrid model proposed by [43] processes features in a hierarchical manner, which may lead to the loss of valuable information in the transition between architectures. These limitations highlight our use of a parallel habitation technique and feature-level fusion to accurately distinguish between two mimic chest diseases, Tuberculosis and Pneumonia, through CXR images. Table 1, Table 2 and Table 3 show a comparative study of the performance of CNN and ViT-based approaches according to the existing literature.

## 3. Methodology

This section presents the working approach in detail, starting with the datasets used as well as the preprocessing and data augmentation phase, and also including the architecture and the different configurations used for the classification process.

### 3.1. The Proposed Approach

In this paper, we incorporated CNNs and ViTs to benefit from the strength of both by designing a hybrid model based on ResNet-50 and ViT-b16, pretrained on the ImageNet dataset in order to combine the spatial feature extraction capability of ResNet-50 with the global contextual understanding of ViT-b16.

The ResNet-50 introduces the concept of residual learning by applying the skip connections that allow the model to focus on incremental improvements rather than complete transformations. The skip connections can be formulated as:

(1) $$H\left(x\right)=F\left(x,\;\left\{W_{i}\right\}\;\right)+x$$

where *H*(*x*) denotes represents the desired underlying mapping, and *F*(*x*) and *x* denote the residual function that captures changes in features through learned weights *W_i_* and the input to the bloc, respectively.

The complete residual block consists of convolutional operations, incorporating batch normalization (BN) and the *Relu* activation function σ as detailed in Equation (2):

(2) $$output=\sigma (BN(conv(\sigma (\;BN(conv\left(x\right))))))$$

The ViT-b16 splits the images into 16 × 16 patches *p_i_* and linearly embeds them into a vector *z_i_* of dimension *d* using a linear projection, which is formulated as:

(3) $$z_{i}=W_{p}\cdot p_{i}+b_{i}$$

where *W_i_* represents the learnable projection matrix and *b* denotes the bias. Next, a learnable position embedding is added to retrain spatial information.

(4) $$z_{i}^{\prime }=z_{i}+Position\;embedding$$

These embedded patches are fed to the transformer block encoder, which consists of 12 encoders, each containing 12 attention heads and a Multi-Layer Perceptron (MLP) block with a Gelu activation function. The self-attention of each head is presented as follows:

(5) $$Attention=\left(Q,\;K,V\right)=softmax\left(\frac{QK^{T}}{\sqrt{d_{k}}}\right)\;V$$

Q, K, and V represent the query, key, and values matrices, respectively, derived from the input through learnable projections.

Finally, a vector with a size of 768 (16 × 16 × 3) is passed through the classification head, which is a single dense layer with a single neuron that indicates Tuberculosis or normal in our case.

(6) $$Output={\sigma (W}_{out}\cdot z^{f}+b_{out})$$

The output is a probability, which ranges between 0 and 1. σ represents the sigmoid activation function, $W_{out}$ is the weight matrix of the final dense layer, $z^{f}$ is the final 768-dimensional feature vector from the ViT-b16 model, and $b_{out}$ represents the bias of term of the final letter.

**Figure 1:**
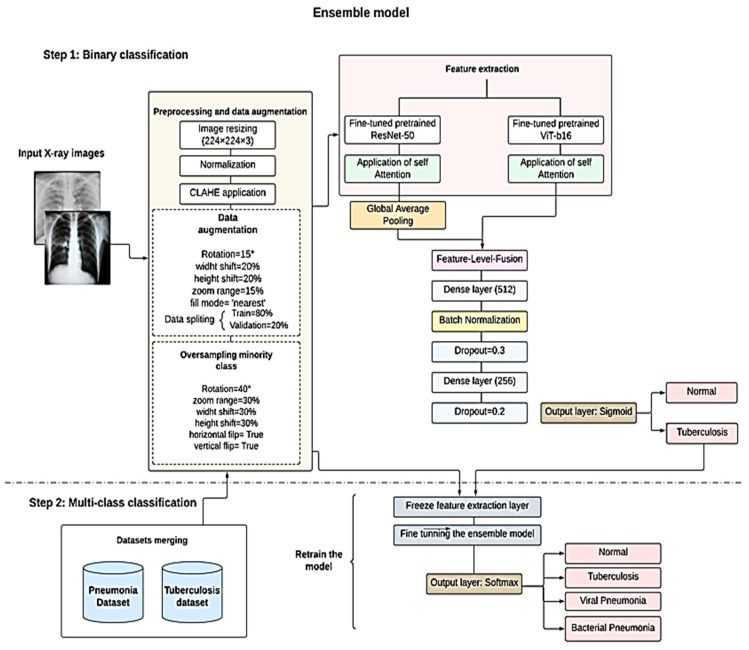
The overall ensemble model architecture.

The input to the entire ensemble model (Figure 1) is an image of 224 × 224 × 3, which is trained end-to-end as a single model by applying feature-level fusion to combine and concatenate the features extracted by each model. This enables the model to optimally combine the features from both streams during training. At this level, a self-attention mechanism was applied in both streams using the attention layer. This allows the model to focus on the most relevant features from each one. The attention layers were followed by a global average pooling to reduce the spatial dimensions, ensuring that the features maps from ResNet-50 match the output shape of ViT-b16. The two models were fine-tuned to maintain the benefits of transfer learning, where the last 10 layers for the ResNet-50 and the last 4 layers for the ViT-b16 are unfrozen, while keeping the earlier layers frozen, preserving the general feature extraction capabilities, while allowing for the adaption to our specific datasets.

The extracted and processed features were passed through two fully connected layers (dense layers) with 512 and 256 neurons, respectively, each one followed by batch normalization and dropout layers, leading to the best generalization and stability, effectively adapting the pretrained features for our task. The final layer represents the output layer for the classification process, which consists of a single neuron with a sigmoid activation function for the binary classification task.

### 3.2. Material and Methods

#### 3.2.1. Dataset

In our study, we used the following publicly available datasets in Kaggle [50,51]. The Tuberculosis chest X-ray dataset is used, which includes two classes (TB-positive and TB-negative). The images are collected by a team of researchers from Qatar and Dhaka (Bangladesh) universities with their Malaysian collaborators. Only 700 TB images are publicly available, against 3500 normal images.

The Pneumonia dataset was obtained from Guangzhou Women’s and Children’s Medical Center, which contains 1344 images of normal cases and 3875 images of Pneumonia cases, devised to 2530 samples of bacterial Pneumonia and 1345 samples of viral Pneumonia. The Tuberculosis dataset was first used for a binary classification to detect Tuberculosis and differentiate between normal cases and Tuberculosis cases. Then, we merged the two datasets for a multi-class classification task to distinguish between viral Pneumonia, bacterial Pneumonia, and Tuberculosis.

#### 3.2.2. Preprocessing and Data Augmentation

**Figure 2:**
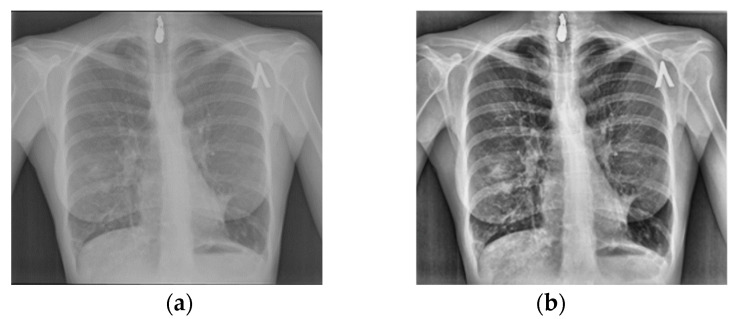
Example of Tuberculosis cases (**a**): the original image; (**b**): the image after the application of CLAHE.

As a start, the images were resized to 224 × 224 × 3. Next, normalization was applied in order to scale the pixels values to a range [0, 1] that is suitable for model training to perform and generalize better to new data by reducing the influence of outliers and ensuring that all features contribute effectively in the learning process. Then, Contrast Limited Adaptive Histogram Equalization (CLAHE) was used with a cliplimit of 3.5 (Figure 2) to improve the local contrast of images, enhancing visibility in lighter and darker regions. This makes the detection of features like keypoints and edges more effective. The improved contrast makes subtle differences in pixel intensities more pronounced, allowing feature detection algorithms to more easily identify and localize important structures.

Afterward, a general data augmentation process was applied to increase the size of the entire dataset and introduce some variability in the training process, which helps to reduce the possibility of overfitting by encouraging the model to learn more general and transferable features. Four geometric transformations were used, namely a rotation of 15°, a horizontal and vertical translation of 20%, and a zoom range of 15%. These transformations offer new samples that encourage the learning of invariant features. Then, we employed a ‘nearest fill’ mode to handle the empty spaces created at the image edges by filling any newly created empty pixels with the value of the nearest valid pixel.

In order to treat the problem of data imbalance, which occurs significantly in both of the two datasets used, we employed an oversampling technique targeted at the minority classes. This strategy involved generating additional samples through the application of more aggressive augmentation, which includes a rotation up to 40°, a zoom range of 30%, horizontal and vertical translations up to 30%, and horizontal and vertical flips. The application of oversampling gives the model more opportunities to learn from minority class samples, which mitigate the risk of developing a bias toward the majority classes.

Finally, the training dataset was randomly split into ‘Train’ and ‘validation’ using the following ratio: 80% and 20%, respectively.

## 4. Experimental Study

### 4.1. Evaluation Metrics

The performance of the ensemble model was evaluated using the following metrics: accuracy (Equation (7)), which gives an overview about the model’s performance across all classes; and recall (Equation (8)), which is better suited for imbalanced datasets by capturing all the instances of the minority classes. This metric is important when the cost of missing positive instance is high. The precision (Equation (9)) metric ensures that the positive predictions made by the model are generally correct. We also used the F1-score metric (Equation (10)), providing a balance between the precision and recall which is useful when both false positives and false negatives are critical. Additionally, the Jaccard score (Equation (11)) and the Dice coefficient (Equation (12)) were also calculated; the first gauges the similarity and diversity of sample sets and illustrates how well the model handles the trade-off between precision and recall, and the second shows the effectiveness of the distinction between the model’s classes.

*Accuracy* (*acc*) = (*TP* + *TN*)/(*TP* + *TN* + *FP* + *FN*) (7)

*Recall* = *TP*/(*TP* + *FN*) (8)

*Precision* = *TP*/(*TP* + *FP*) (9)

*F1-score* = (2 × *Precision* × *Recall*)/(*Precision* + *Recall*) (10)

*Jaccard score* = (*Precision* × *Recall*)/*Precision* + *Recall* − (*Precision* × *Recall*) (11)

*Dice coefficient* = 2 × *TP*/(2 × *TP* + *FP* + *FN*) (12)

where true positives (TPs) indicate the model’s ability to correctly detect patients with the disease, which is crucial for timely and accurate treatment. True negatives (TNs) represent the model’s ability to correctly identify patients without anomalies. False positives (FPs) occur when the model incorrectly identifies a disease that is not present. Conversely, false negatives (FNs) occur when the model fails to detect the presence of a disease, which is an unacceptable mistake as it delays treatment and potentially worsens patient outcomes.

### 4.2. Model Training and Experimental Setup

The model was first trained for a binary classification task using the Tuberculosis dataset, with TensorFlow version 2.10.1 on a GeForce 1050 Ti graphic card with dedicated memory. We also used Google Collab Pro for more performant GPUs, namely the T4 and A100 GPUs.

The choice of the *ResNet-50* was confirmed after testing different fine-tuned pretrained CNN models such as VGG-16, DenseNet-121, etc., where the ResNet-50 produced a better result. For the ViT, we only tested the base model instead of the large ViT and huge ViT models, due to the computation costs and time-related efforts with different patch sizes such as 32 × 32, as the 16 × 16 patch size enhances the model’s performance.

We employed the *RectifiedAdam optimizer* with an initial learning rate of 1 × 10^−4^ to obtain a more accurate and generalizable deep neural network by rectifying the variance of the adaptive learning rate; we also used binary cross entropy as a loss function. The model was trained for 30 epochs with a batch size of 16. During this training we implemented early stopping based on the validation F1-score to prevent overfitting. The F1-score considers both false positives and false negatives, which is critical in the medical context. We introduced callbacks to automatically adjust training parameters and to save the best model, ensuring the retraining of the optimal model.

The ensemble model was extended to perform multi-class classification for the distinction between Tuberculosis, viral Pneumonia, bacterial Pneumonia, and normal cases, based on the knowledge gained during the binary classification.

Firstly, we merged the dataset of Tuberculosis and the dataset of Pneumonia, as is mentioned above. This merging caused a data imbalance. To address this issue, we kept the data augmentation techniques, including the oversampling of minority classes; then, we implemented the class weights method to give more importance to underrepresented classes during training. We changed the classification head by replacing the output layer with another that contained four neurons, each corresponding to one of the four classes, combined with softmax activation function.

We maintained our original training and optimization methods. However, we changed the loss function with focal categorical entropy, which is particularly useful for multi-class classification and imbalanced data; we also minimized the learning rate value to 1 × 10^−5^ to facilitate fine-tuning without altering the pre-learned features. In this step, the model trained only for 15 epochs due to computation limits.

## 5. Results

The proposed hybrid workflow was designed by combining the capabilities of both ResNet-50 and ViT-b16. The model was trained within two steps. In the first step, a binary classification for Tuberculosis detection was applied. In the second step, we performed a multi-class classification to effectively distinguish between Tuberculosis, bacterial Pneumonia, and viral Pneumonia. The model’s performance was evaluated over three scenarios: testing the performance of individual CNNs, individual ViTs, and hybrid model-based CNN-ViT. Table 4, Table 5, Table 6 and Table 7 present the obtained results according to these scenarios.

**Figure 3:**
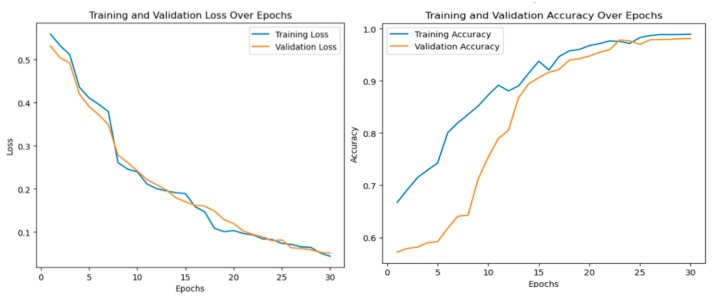
Ensemble model performance in terms of loss and accuracy for the binary classification.

**Figure 4:**
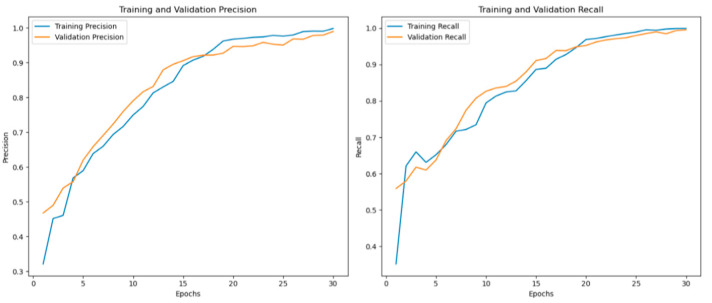
Ensemble model performance in terms of precision recall for the binary classification.

For the binary classification step, the hybrid model achieved a performance of 98.97% in terms of accuracy, 99.87% in terms of precision, 99.91% in terms of recall (Figure 3 and Figure 4) and 99.08% in terms of F1-score. The ensemble model outperforms both individual CNNs and ViTs.

**Figure 5:**
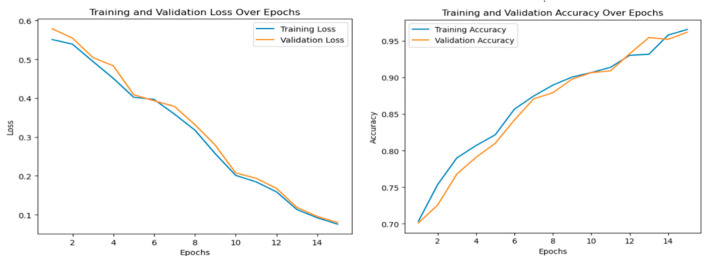
Ensemble model performance in terms of loss and accuracy for the multi-class classification.

**Figure 6:**
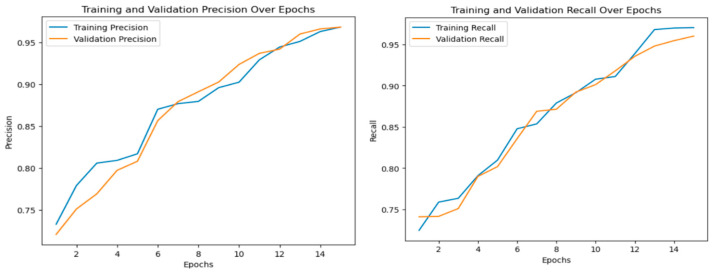
Ensemble model performance in terms of precision and recall for the multi-class classification.

For the multi-class classification step, the hybrid model achieved acceptable performance with an accuracy of 96.18%, a precision of 96.8%, a recall of 96% (Figure 5 and Figure 6), and an F1-score of 96.4%.

## 6. Discussion

In this study, we presented a novel hybrid model based on CNN and ViT for Tuberculosis detection as a binary classification task, and the distinction between Tuberculosis, bacterial Pneumonia, viral Pneumonia, and Normal cases.

In binary classification, the obtained results proved that our hybrid model outperforms the standalone CNNs and ViTs. The balanced precision and recall across all classes (Table 1 and Table 2) indicates that the model achieves a consistent performance for the underrepresented classes, which is crucial in the medical context. The model’s high F1-score suggest its stability and potential application in real-world clinical environments, especially in regions with limited access to radiology experts.

Our model exhibits competitive performance compared to several state-of-the-art studies. For instance, the obtained accuracy of 98.97% for Tuberculosis detection surpasses the hybrid EfficientNet-ViT model proposed by [52] that achieved an accuracy of 97.92%, which proves the robustness of our fine-tuning technique for the ensemble model.

In the case of multi-class classification, ViT-b16 slightly outperformed the hybrid model, which was attributed to the increased complexity of the task. Furthermore, we were not able to train the model for more than 15 epochs due to computation capacity limitations.

Table 8 represents a detailed comparison between our approach and state-of-the-art models that combine CNNs with ViT models. The results indicate that our hybrid model outperforms these models in both binary classification and multi-class classification.

## 7. Conclusions

In the last decade, chest disease detection-based on chest X-ray images using deep learning models has been an active area of research. Chest X-ray image interpretation can be challenging due to the overlap of anatomical structures, the similarities of symptoms between diseases, and the shortage of radiologists. In this study, we proposed a hybrid model based on ResNet-50 and ViT-b16 for Tuberculosis detection and distinction between Tuberculosis, viral Pneumonia, and bacterial Pneumonia. This approach achieved promising results with an accuracy of 98.97% and an F1-score of 99.08% for binary classification, and an accuracy of 96.4% and an F1-score of 96.18% for multi-class classification. The hybrid model outperformed a lot of state-of-the-art models for both binary classification and multi-class classification.

Despite the promising results achieved by our hybrid model, the limitation of computational resources prevented us from training the model for more than 15 epochs in the case of multi-class classification. With additional computational capacities, it is likely that training for more epochs could lead to further performance improvements. Our future goal is to develop a more computationally efficient version of the model for potential deployment on resource-constrained devices, as well as a model that generalizes efficiently and stably using different datasets.

## Figures and Tables

**Table 1:** Performance comparison of CNN-based approaches for chest X-ray classification.

Authors	Approach	Pathology	Classification Method	Performance
[11]	LightWeight CNN	COVID-19, Pneumonia Pleural effusion, Lung opacity, Cardiomegaly	Multi-class classification	Accuracy = 80%
		Pneumonia, Normal	Binary classification	Accuracy = 97.94%
[12]	LightWeight CNN	Lung opacity, Fibrosis, Pneumonia, COVID-19, Tuberculosis	Multi-class classification	Accuracy = 98.56%
[18]	INFO-MGAN for segmentation + Novel handcrafted features with DCNNs	COVID-19, Lung cancer, Atlectasis, Consolidation lung, Tuberculosis, Pneumothorax, Edema, Pneumonia, Pleural thickening	Multi-class classification	Accuracy = 98.20%
[20]	Yolo V5 for anomalies localization ResNet-50 for classification	Chest abnormalities	Multi-class classification	F1-score = 76%
[22]	Six stacked pretrained CNNs	COVID-19 with X-ray images and Computer Tomography (CT scan) images	Binary classification	Accuracy ≈ 99%
	ECA-Net + EfficientNet V2			
[17]	Fine-tuned CNN	Viral Pneumonia, Bacterial Pneumonia, Normal	Binary classification	Accuracy = 92.8%
[14]	Fine-tuned VGG-19	Lung cancer, Lung opacity, COVID-19, Pneumonia, Tuberculosis	Multi-class classification	Accuracy = 93.75%

**Table 2:** Performance comparison of ViT-based approaches for chest X-ray classification.

Authors	Approach	Pathology	Classification Method	Performance
[27]	Volo	COVID-19	Binary classification	Accuracy = 99.67%
[30]	Fine-tuned ViT	COVID-19	Binary classification	Accuracy = 96%
[32]	ViT-b32	COVID-19, Pneumonia, Normal	Multi-class classification	Accuracy = 97.61%
[33]	ViT-b32	Normal Lung, Diseased Lung, COVID-19	2-Step Binary classification	Accuracy = 95.36%
[34]	Pretrained Swin Transformer	14 chest diseases from the chest-14 dataset	Multi-class classification	AUC = 81%
[36]	Pretrained ViT	Pneumonia, Normal	Binary classification	F1-score = 86%
		14 chest diseases from the Chexpert dataset (Irvin et al., 2019 [37])	Multi-class classification	F1-score = 59%
[38]	Modified ViT	Heart diseases and lung diseases	Multi-class classification	AUC = 95.13%
			Binary classification	AUC ≈ 99%
[39]	U-Net for lung segmentation ViT for classification	COVID-19	Binary classification	AUC > 95%
[40]	Adapted ViT	14 chest diseases from chest-14 dataset [49] (Wang, 2017)	Multi-class classification	Accuracy = 83.4%
[25]	PneuNet	Pneumonia, COVID-19	Multi-class classification	Accuracy = 94.96%

**Table 3:** Comparative analysis of hybrid CNN-ViT models for chest disease detection.

Authors	Approach	Pathology	Classification Method	Performance
[42]	Enhanced Vit: Parallel CNN and ViT	COVID-19, Tuberculosis, and Pneumonia, using different datasets	Multi-class classification	93.5% ≤ Recall ≤ 100%
[43]	Ensemble CNNs + ViT	Pneumonia	Binary classification	Accuracy = 98.01%
[28]	EfficientNet + ViT	Tuberculosis	Binary classification	Accuracy = 97.72%
[44]	VGG-16 + CoorAttention	Tuberculosis, using merged datasets	Binary classification	Accuracy = 92.73%
[45]	CNN-ViT	Tuberculosis, using lateral and frontal CXR images	Binary classification	Accuracy = 91%
[47]	ResfEANet	Tuberculosis	Binary classification	Accuracy = 97.95%
[48]	Hydra-ViT	14 chest diseases from the chest-14 dataset	Multi-class classification	AUC = 83.8%

**Table 4:** Confusion matrix for binary classification.

	Normal	Tuberculosis
Normal	2444	6
Tuberculosis	2	487

**Table 5:** Confusion matrix for multi-class classification.

	Normal	Tuberculosis	Viral Pneumonia	Bacterial Pneumonia
Normal	4653	68	43	80
Tuberculosis	8	672	7	13
Viral Pneumonia	14	18	1295	18
Bacterial Pneumonia	38	20	32	2440

**Table 6:** The classification evaluation performance of the tested deep learning models for binary classification.

Pretrained Models	Accuracy	Precision	Recall	F1-Score	Jaccard Score	Dice Coefficient
VGG-16	0.7756	0.7698	0.3309	0.4628	0.3713	0.4428
DenseNet-121	0.8299	0.8098	0.8355	0.8224	0.7198	0.7100
Xception	0.8754	0.8960	0.8934	0.8947	0.8063	0.7988
ResNet-50	0.9115	0.9210	0.9077	0.9114	0.8464	0.8137
ViT-b16	0.9418	0.9367	0.9549	0.9457	0.8964	0.8511
ViT-b32	0.8909	0.9023	0.8809	0.8915	0.7890	0.8095
Ensemble model	0.9897	0.9987	0.9991	0.9908	0.9838	0.8914

**Table 7:** The classification evaluation performance of the tested deep learning models for multi-class classification.

Pretrained Models	Accuracy	Precision	Recall	F1-Score	Jaccard Score	Dice Coefficient
VGG-16	0.5518	0.5657	0.5187	0.5412	0.9756	0.6821
DenseNet-121	0.9043	0.8945	0.8898	0.8921	0.8064	0.7508
Xception	0.8514	0.8438	0.8499	0.8468	0.7543	0.8224
ResNet-50	0.9365	0.9411	0.9387	0.9384	0.8839	0.8854
ViT-b16	0.9725	0.9710	0.9766	0.9738	0.9245	0.8975
ViT-b32	0.9156	0.8965	0.9076	0.9020	0.8623	0.8813
Ensemble model	0.9618	0.9680	0.9600	0.9640	0.9032	0.9124

**Table 8:** Comparative analysis of hybrid CNN-ViT models for chest disease detection.

Authors	Approach	Achieved Result	Authors	Approach	Achieved Results
[42]	Enhanced Vit: Parallel CNN and ViT	93.5% ≤ recall ≤ 100%	[44]	VGG-16 + CoorAttention	Accuracy = 92.73%
[43]	Ensemble CNNs + ViT	Accuracy = 98.01%	[47]	ResfEANet	Accuracy = 97.95%
[28]	EfficientNet + ViT	Accuracy = 97.72%	[45]	CNN-ViT	Accuracy = 91%
[48]	HydraViT	AUC = 83.8%	Our hybrid model	ResNet50-ViTb16	Accuracy = 98.97% for binary classification

## Data Availability

Data will be available on request.

## References

[1] Levine S.M., Marciniuk D.D. (2022). Global Impact of Respiratory Disease. Chest.

[2] World Health Organization. https://www.who.int/news-room/fact-sheets/detail/chronic-obstructive-pulmonary-disease-(copd).

[3] World Health Organization Tuberculosis. https://www.who.int/news-room/fact-sheets/detail/tuberculosis.

[4] Schildknecht K.R., Pratt R.H., Feng P.-J.I., Price S.F., Self J.L. (2023). Tuberculosis—United States, 2022. MMWR. Morb. Mortal. Wkly. Rep..

[5] World Health Organization Global Tuberculosis Report 2024. https://www.who.int/teams/global-tuberculosis-programme/tb-reports/global-tuberculosis-report-2024.

[6] UNICEF Pneumonia in Children: What You Need to Know. https://www.unicef.org/stories/childhood-pneumonia-explained#:~:text=Everyyear%2Cpneumoniaclaimsthe,ofthesedeathsarepreventable.

[7] Qin C., Yao D., Shi Y., Song Z. (2018). Computer-aided detection in chest radiography based on artificial intelligence: A survey. Biomed. Eng. Online.

[8] Vaswani A., Shazeer N., Parmar N., Uszkoreit J., Jones L., Gomez A.N., Kaiser Ł., Polosukhin I. (2017). Attention is all you need. Advances in Neural Information Processing Systems.

[9] Dosovitskiy A., Beyer L., Kolesnikov A., Weissenborn D., Zhai X., Unterthiner T., Dehghani M., Minderer M., Heigold G., Gelly S. An Image Is Worth 16x16 Words: Transformers for Image Recognition at Scale. Proceedings of the ICLR 2021—9th International Conference on Learning Representations.

[10] Chernyavskiy A., Ilvovsky D., Nakov P. (2021). Transformers: “The End of History” for Natural Language Processing?. Lecture Notes in Computer Science (Including Subseries Lecture Notes in Artificial Intelligence and Lecture Notes in Bioinformatics).

[11] Nahiduzzaman M., Islam M.R., Hassan R. (2023). ChestX-Ray6: Prediction of multiple diseases including COVID-19 from chest X-ray images using convolutional neural network [Formula presented]. Expert Syst. Appl..

[12] Sanida T., Dasygenis M. (2024). A novel lightweight CNN for chest X-ray-based lung disease identification on heterogeneous embedded system. Appl. Intell..

[13] Hedhoud Y., Mekhaznia T., Amroune M. (2023). An improvement of the CNN-XGboost model for pneumonia disease classification. Pol. J. Radiol..

[14] Alshmrani G.M.M., Ni Q., Jiang R., Pervaiz H., Elshennawy N.M. (2023). A deep learning architecture for multi-class lung diseases classification using chest X-ray (CXR) images. Alex. Eng. J..

[15] Guefrechi S., Jabra M.B., Ammar A., Koubaa A., Hamam H. (2021). Deep learning based detection of COVID-19 from chest X-ray images. Multimed. Tools Appl..

[16] Ibrahim A.U., Ozsoz M., Serte S., Al-Turjman F., Yakoi P.S. (2024). Pneumonia Classification Using Deep Learning from Chest X-ray Images During COVID-19. Cognit. Comput..

[17] Kermany D.S., Goldbaum M., Cai W., Valentim C.C.S., Liang H., Baxter S.L., McKeown A., Yang G., Wu X., Yan F. (2018). Identifying Medical Diagnoses and Treatable Diseases by Image-Based Deep Learning. Cell.

[18] Malik H., Anees T., Chaudhry M.U., Gono R., Jasinski M., Leonowicz Z., Bernat P. (2023). A Novel Fusion Model of Hand-Crafted Features With Deep Convolutional Neural Networks for Classification of Several Chest Diseases Using X-ray Images. IEEE Access.

[19] Hu X., Chen J., Chen Y. (2024). RegMamba: An Improved Mamba for Medical Image Registration. Electronics.

[20] Pham V.T.N., Nguyen Q.C., Nguyen Q.V. (2023). Chest X-Rays Abnormalities Localization and Classification Using an Ensemble Framework of Deep Convolutional Neural Networks. Vietnam J. Comput. Sci..

[21] Nguyen H.Q., Lam K., Le L.T., Pham H.H., Tran D.Q., Nguyen D.B., Le D.D., Pham C.M., Tong H.T.T., Dinh D.H. (2022). VinDr-CXR: An open dataset of chest X-rays with radiologist’s annotations. Sci. Data.

[22] Huang M.L., Liao Y.C. (2023). Stacking Ensemble and ECA-EfficientNetV2 Convolutional Neural Networks on Classification of Multiple Chest Diseases Including COVID-19. Acad. Radiol..

[23] Tanzi L., Audisio A., Cirrincione G., Aprato A., Vezzetti E. (2022). Vision Transformer for femur fracture classification. Injury.

[24] Shou Y., Meng T., Ai W., Xie C., Liu H., Wang Y. (2022). Object Detection in Medical Images Based on Hierarchical Transformer and Mask Mechanism. Comput. Intell. Neurosci..

[25] Wang C., Jin Y., Liang J., Yang Y.H., Nie S., Hai Y. Modif-SegUnet: Innovatively Advancing Liver Cancer Diagnosis and Treatment through Efficient and Meaningful Segmentation of 3D Medical Images. Proceedings of the 2023 IEEE International Conference on Bioinformatics and Biomedicine, BIBM 2023.

[26] Liu Z., Lin Y., Cao Y., Hu H., Wei Y., Zhang Z., Lin S., Guo B. Swin Transformer: Hierarchical Vision Transformer using Shifted Windows. Proceedings of the IEEE International Conference on Computer Vision.

[27] Liu C., Yin Q. (2021). Automatic diagnosis of COVID-19 using a tailored transformer-like network. J. Phys. Conf. Ser..

[28] Cohen J.P., Morrison P., Dao L., Roth K., Duong T., Ghassem M. (2020). COVID-19 Image Data Collection: Prospective Predictions are the Future. Mach. Learn. Biomed. Imaging.

[29] Chowdhury M.E.H., Rahman T., Khandakar A., Mazhar R., Kadir M.A., Mahbub Z.B., Islam K.R., Khan M.S., Iqbal A., Emadi N.A. (2020). Can AI Help in Screening Viral and COVID-19 Pneumonia?. IEEE Access.

[30] Chetoui M., Akhloufi M.A. (2022). Explainable Vision Transformers and Radiomics for COVID-19 Detection in Chest X-rays. J. Clin. Med..

[31] SIIM-FISABIO-RSNA SIIM-FISABIO-RSNA COVID-19 Detection Challenge. https://siim.org/research-journal/siim-machine-learning-challenges/covid-19-kaggle-challenge/.

[32] Krishnan K.S., Krishnan K.S. Vision Transformer based COVID-19 Detection using Chest X-rays. Proceedings of the IEEE International Conference on Signal Processing, Computing and Control.

[33] Than J.C.M., Thon P.L., Rijal O.M., Kassim R.M., Yunus A., Noor N.M., Then P. Preliminary Study on Patch Sizes in Vision Transformers (ViT) for COVID-19 and Diseased Lungs Classification. Proceedings of the 2021 IEEE National Biomedical Engineering Conference (NBEC).

[34] Taslimi S., Taslimi S., Fathi N., Salehi M., Rohban M.H. (2022). SwinCheX: Multi-label classification on chest X-ray images with transformers. arXiv.

[35] National Institutes of Health Chest X-Ray—Chris Crawford NIH Chest X-Rays Dataset. Kaggle. https://www.kaggle.com/datasets/nih-chest-xrays/data.

[36] Usman M., Zia T., Tariq A. (2022). Analyzing Transfer Learning of Vision Transformers for Interpreting Chest Radiography. J. Digit. Imaging.

[37] Irvin J., Rajpurkar P., Ko M., Yu Y., Ciurea-Ilcus S., Chute C., Marklund H., Haghgoo B., Ball R., Shpanskaya K. (2019). CheXpert: A large chest radiograph dataset with uncertainty labels and expert comparison. Proc. AAAI Conf. Artif. Intell..

[38] Nasser A.A., Akhloufi M.A. (2023). Deep Learning Methods for Chest Disease Detection Using Radiography Images. SN Comput. Sci..

[39] Park S., Kim G., Oh Y., Seo J.B., Lee S.M., Kim J.H., Moon S., Lim J.-K., Ye J.C. (2022). Multi-task vision transformer using low-level chest X-ray feature corpus for COVID-19 diagnosis and severity quantification. Med. Image Anal..

[40] Huang L., Ma J., Yang H., Wang Y. (2024). Research and implementation of multi-disease diagnosis on chest X-ray based on vision transformer. Quant. Imaging Med. Surg..

[41] Wang T., Nie Z., Wang R., Xu Q., Huang H., Xu H., Xie F., Liu X.-J. (2023). PneuNet: Deep learning for COVID-19 pneumonia diagnosis on chest X-ray image analysis using Vision Transformer. Med. Biol. Eng. Comput..

[42] Okolo G.I., Katsigiannis S., Ramzan N. (2022). IEViT: An enhanced vision transformer architecture for chest X-ray image classification. Comput. Methods Programs Biomed..

[43] Ukwuoma C.C., Qin Z., Belal Bin Heyat M., Akhtar F., Bamisile O., Muaad A.Y., Addo D., Al-antari M.A. (2023). A hybrid explainable ensemble transformer encoder for pneumonia identification from chest X-ray images. J. Adv. Res..

[44] Xu T., Yuan Z. (2022). Convolution Neural Network With Coordinate Attention for the Automatic Detection of Pulmonary Tuberculosis Images on Chest X-Rays. IEEE Access.

[45] Rajaraman S., Zamzmi G., Folio L.R., Antani S. (2022). Detecting Tuberculosis-Consistent Findings in Lateral Chest X-Rays Using an Ensemble of CNNs and Vision Transformers. Front. Genet..

[46] Bustos A., Pertusa A., Salinas J.-M., de la Iglesia-Vayá M. (2020). PadChest: A large chest X-ray image dataset with multi-label annotated reports. Med. Image Anal..

[47] Ejiyi C.J., Qin Z., Nnani A.O., Deng F., Ejiyi T.U., Ejiyi M.B., Agbesi V.K., Bamisile O. (2024). ResfEANet: ResNet-fused External Attention Network for Tuberculosis Diagnosis using Chest X-ray Images. Comput. Methods Programs Biomed. Updat..

[48] Öztürk Ş., Turalı M.Y., Çukur T. (2023). HydraViT: Adaptive Multi-Branch Transformer for Multi-Label Disease Classification from Chest X-ray Images. arXiv.

[49] Wang X., Peng Y., Lu L., Lu Z., Bagheri M., Summers R.M. ChestX-ray8: Hospital-scale chest X-ray database and benchmarks on weakly-supervised classification and localization of common thorax diseases. Proceedings of the 30th IEEE Conference on Computer Vision and Pattern Recognition, CVPR 2017.

[50] Rahman T., Khandakar A., Kadir M.A., Islam K.R., Islam K.F., Mazhar R., Hamid T., Islam M.T., Kashem S., Mahbub Z.B. (2020). Reliable tuberculosis detection using chest X-ray with deep learning, segmentation and visualization. IEEE Access.

[51] Chest X-Ray Images (Pneumonia) Kaggle. https://www.kaggle.com/datasets/paultimothymooney/chest-xray-pneumonia/data.

[52] Duong L.T., Le N.H., Tran T.B., Ngo V.M., Nguyen P.T. (2021). Detection of tuberculosis from chest X-ray images: Boosting the performance with vision transformer and transfer learning. Expert Syst. Appl..

